# Pharmacological Effects of Selected Medicinal Plants and Vitamins Against COVID-19

**DOI:** 10.17303/jfn.2021.7.202

**Published:** 2021-07-12

**Authors:** Clement G Yedjou, Sylvianne Njiki, Juliet Enow, Otto Ikome, Lekan Latinwo, Richard Long, Pierre Ngnepieba, Richard A Alo, Paul B Tchounwou

**Affiliations:** 1 Department of Biological Sciences, College of Science and Technology, Florida Agricultural and Mechanical University, 1610 S. Martin Luther King Blvd, Tallahassee, United States; 2 Department of Biology, College of Science, Engineering and Technology, Jackson State University, 1400 Lynch Street, Box 18750, Jackson, United States; 3 Department of Behavioral and Environmental Health. School of Public Health, Jackson State University, 350 W. Woodrow Wilson Drive, Jackson, United States; 4 Department of Mathematics, College of Science and Technology, Florida Agricultural and Mechanical University, 1610 S. Martin Luther King Blvd, Tallahassee, United States; 5 Department of Computer and Information Science, College of Science and Technology, Florida Agricultural & Mechanical University, 1610 S. Martin Luther King Blvd, Tallahassee, United States

**Keywords:** Medicinal Plants, Vitamin C, Vitamin D, Allium Sativum, Curcumin, Nigella Sativa, Zingiber Officitale, COVID-19, Antiviral Activity, Pharmacological Effects

## Abstract

The coronavirus disease 2019 (COVID-19) is caused by the severe acute respiratory syndrome coronavirus-2 (SARS-CoV-2). It is a serious disease that has caused multiple deaths in various countries in the world. Globally, as of May 23, 2021, the total confirmed cases of COVID-19 have reach 166,346,635 with a total of 3,449,117 deaths. Several recent scientific studies have shown that medicinal plants and vitamins can benefit and improve the health of COVID-19 patients. However, the benefits of medicinal plants and vitamins in the treatment of COVID-19 remain unproven. Therefore, the objective of this article is to expounds the benefits of using medicinal plants (*Allium sativum*, curcumin, *Nigella sativa*, *Zingiber officitale*) and vitamins (vitamin C and vitamin D) that possess the antiviral properties for the prevention and/or control of COVID-19. To reach our objective, we searched scientific databases of ongoing trials in the Centers for Disease Control and Prevention websites, PubMed Central, Medline databases, and Google Scholar websites. We also searched databases on World Health Organization International Clinical Trials Registry Platform to collect relevant papers. We found that all of the selected medicinal plants and vitamins possess antiviral activities, and their individual intake shows promise for the prevention and/or control of COVID-19. We conclude that, the selected medicinal plants and vitamins possess anti-viral properties that are more likely to prevent and/or disrupt the SARS-CoV-2 replication cycle, enhance the human immune system and promote good health.

## Introduction

The current major health crisis in the world is the third serious Coronavirus outbreak in less than 20 years, following severe acute respiratory syndrome (SARS) in 2002–2003 and Middle East Respiratory Syndrome (MERS) in 2012. The coronavirus disease including COVID-19 caused by the SARS coronavirus-2 (SARS-coV-2) may present a spectrum of disease severity between flu –like symptoms to death via acute respiratory distress syndrome [1,2]. The novel coronavirus disease 2019 (COVID-19) is caused by the severe acute respiratory syndrome coronavirus-2 (SARS-CoV-2). The virus is well-known to cause severe bilateral pneumonia and acute respiratory distress syndrome (ARDS) that may lead to breathing difficulties and can be managed by mechanical ventilation and intense care unit [3,4]. It is usually transmitted through the respiratory tract via droplets and a healthy person can be infected with COVID-19 virus by touching contaminated surface and then used the hands to touch the nose, eye, or mouth without washing the hands [5].

The current targeted treatment for COVID-19 approved by Food and Drug Administration (FDA) is “Remdevisir”, making Remdevisir the first-line treatment option for COVID-19 treatment. It acts as an inhibitor of RNA polymerase and its characteristics and pharmacokinetics have been studied in MERS-CoV and SARS-CoV infections. A recent study demonstrated that Remdevisir, an antiviral drug is effective against COVID-19 in the epithelial cells of human airways and has virologic and clinical efficacy in non-human primates [6]. However, Remdevisir is not recommended for patients who require mechanical ventilation because there are limited medical data showing its benefit on the treatment of advanced stage of the disease [7–10]. Other drugs used include anti-viral drug such as ribavirin [11], antimalarial drugs (chloroquine and Hydroxychloroquine) [12], and a combination of retroviral drugs (Ritonavir/lopinavir) [13]. Furthermore, two combination drugs including bamlanivimab plus etesevimab and casirivimab plus imdevimab are available through FDA Emergency Use Authorization (EUAs) for the treatment of mild to moderate COVID-19 in nonhospitalized with laboratory confirmed COVID-19 infection who have higher prevalence for progressing to severe disease. Clinical management of COVID-19 can be achieved through prevention of the infection, supportive care, and oxygen and mechanical ventilatory support. Other preventive measures include social distancing, hand washing, shelter-in-place, environmental hygiene, and wearing face masks [14,15]. Several recent scientific studies have shown that medicinal plants and vitamins can benefit and improve the health of COVID-19 patients. Therefore, people are seeking preventive ways to protect themselves from the virus. One such preventive way that is well-documented in the literature and often in the news is the use of medicinal plants and vitamins. Traditionally, medicinal plants have been used for improving human health and preventing and/or treating many diseases. Experimental trials have documented the antioxidant, anti-fungal, anti-microbial, anti-cancer, and anti-inflammatory activities of medicinal plants and vitamins against COVID-19. Therefore, it is logical to use medicinal plants and vitamins to prevent and control the virus that causes COVID-19. This article’s objective is to expound on the benefits of using medicinal plants (*Allium sativum*, curcumin, *Nigella sativa*, *Zingiber officitale*) and vitamins (vitamins C and D) that have an antiviral property for the prevention and/or control of COVID-19. In the present study, we selected to investigate *Allium sativum*, curcumin, *Nigella sativa*, *Zingiber officitale*, and vitamins (vitamins C and D) because they share in common eight pharmacological effects and have the most potential antiviral activities against COVID-19.

## Approaches

A broad systematic literature search was conducted in four electronic databases including governmental agency (WHO, CDC) websites, PubMed Central database, Medline database, and Google Scholar websites. We collected publications assessing the antiviral activities and other pharmacological properties of medicinal plants (*Allium sativum*, curcumin, *Nigella sativa*, and *Zingiber officitale*) and vitamins (vitamin C/ascorbic acid and vitamin D) for the prevention and/or control of COVID-19 and we studied peer-reviewed articles published between 2014 and 2021.

## Results and Discussions

During the course of this work, we found that *Allium sativum* (garlic), ascorbic acid (vitamin C), curcumin, *Nigella sativa* (black seed), vitamin D, and *Zingiber officitale* (ginger) share eight (8) commons pharmacological effects including antibacterial, anticancer, anti-inflammatory, immunomodulatory, antioxidant, antifungal, antimutagenic, and antiviral activities. They contain novel therapeutic effects for the prevention and treatment of different human diseases. Vitamin C, an essential micronutrient for humans and free radical scavenger, was used in 2003 for SARS-CoV-1 outbreak as a nonspecific treatment for several respiratory tract infections [16]. Studies showed that vitamin D reduces the risk of common cold and infections through three mechanisms including a physical barrier, cellular natural immunity, and adaptive immunity [17]. A recent study has indicated that garlic and its derivatives fight the coronavirus in the same way as curcumin fights some viruses by preventing the coronavirus from invading cells and stopping the viral replication [18]. Other derivative therapeutic agents from medicinal plants have been reported to inhibit the entry and replication of several coronaviruses. For examples, 3-methylbut-2-enyl)-3′,4,7-trihydroxyflavane (flavonoid) derivative of *Broussonetia papyrifera* inhibits coronavirus proteases enzyme of MERS_CoV virus [19], 4 –Hydroxychalcone (flavonoid) derivative of *Cinnamomum spp* inhibits viral replication of HCoV-NL63 virus and MERS_CoV virus [20,21]. *Echinacea purpurea* inhibits viral replication of SARS-CoV virus, MERS-CoV virus, and HCoV-229E virus [22]. *Gentiana scabra* inhibits viral replication and enzymatic activity of SARS-CoV virus [23]. The potential antiviral activities of *Allium sativum* (garlic), ascorbic acid (vitamin C), curcumin, *Nigella sativa* (black seed), vitamin D, and *Zingiber officitale* (ginger) against different types of coronaviruses could be used to prevent and/or control COVID-19.

### Pharmacological Effects of Ascorbic Acid (Vitamin C) against COVID-19

Vitamin C is an essential compound required in human metabolism. It possesses antioxidant, immunomodulatory, and anti-microbial effects [24]. Studies have shown that vitamin C is an effective immune booster that helps in the prevention of colds [25]. The COVID-19, though not a typical cold/flu virus may partially or fully respond to this mode of treatment. According to Rosetti et al., [26], parenteral administration of vitamin C is an effective and economical treatment plan for the COVID-19 virus. A study by Chiscano-Camón et al., [27], found that among critically ill COVID-19 patients in the intensive care unit (ICU), their plasma levels of vitamin C were undetectable or very low. Another study by Hemilla and Stalker [28,29] found that the administration of vitamin C reduced the stay of patients in the ICU by 8.6% and the duration of patients on mechanical ventilators by 18.2%. This is relevant in the fight against COVID-19 as most critically ill patients end up in the ICU and/or having to use ventilators. Hence, administration of vitamin C could be a means to improve overall health outcomes for patients with COVID-19. A recent report of 17 COVID-19 patients who received 1 g of intravenous vitamin C every 8 hours for 3 days reported a mortality rate of 12% with an 18% rate of intubation and mechanical ventilation and a significant decrease in inflammatory markers, including ferritin and D-dimer, and a trend towards decreasing FiO2 requirements [30]. Also, another report shows unexpected patient recovery following high doses of vitamin C administered intravenously [31]. Findings from these two reports provide the feasibility of using vitamin C to prevent and/or control COVID-19.

### Pharmacological Effects of Allium sativum (garlic) against COVID-19

Garlic (*Allium sativum* L.) is among the oldest cultivated plant, a bulbous herbaceous plant that belongs to the Ama-ryllidaceae family. On the market, there are several formulations of garlic in addition to fresh bulbous garlic, including extracts, capsules, essentials oils, and garlic powder. Garlic has been used since ancient times for culinary purposes in addition to its therapeutic effect [32]. Its medicinal properties have also been used in the treatment of human diseases. The beneficial effects of this old drug include its anti-inflammatory, immunomodulatory, immunostimulatory, cardio protective, hypoglycemic, antioxidant, antibiotic, antifungal, antimicrobial, antiseptic, anticancer, and antiviral activities [33–35]. Recent reports identified garlic and its numerous compounds including s-allyl cysteine(SAC), alli-in, and dially thiosulfafonate (allicin) as promising candidates for potentially improving the immune system [36,37]. Garlic is one of the impressive enhancers of the body’s immune system function because it stimulates natural killer cells (NK cells), macrophages, lymphocytes, eosinophils, and dendritic cells (DCs) through modulation of cytokine secretion, immunoglobulin synthesis, phagocytosis, and macrophages activation [38,39]. A short-term garlic extract supplementation significantly increases T lymphocytes particularly CD4+ and CD8+T cells promoting cellular immune system [40,41]. These immunological markers are known to be at considerably lower levels and related to mortality in nearly all patients with SARS-CoV-2 infection [41,42]. For instance, a study conducted in Wuhan, China, on 452 patients with COVID-19 reported that both helper T cells and suppressor T cells in patients with COVID-19 were below normal levels, and even lower level of helper T cells in severe group [43]. Clinical trials have shown the antiviral effects of garlic against viral cold and flu infections [44], acute respiratory viral infections and immune actions against recalcitrant multiple common warts (RMCW) [45,46]. Furthermore, preclinical data demonstrated that garlic, and its organosulfur compounds (OSCs) have potential antiviral activity against different human, animal, and plant pathogenic viruses through blocking viral entry into host cells, inhibiting viral RNA polymerase, reverse transcriptase, DNA synthesis and immediate-early gene 1(IEG1) transcription, as well as downregulating the extracellular-signal-regulated kinase (ERK)/mitogen activated protein kinase (MAPK) signaling pathway [47].

### Pharmacological Effects of Curcumin against COVID-19

Curcumin is the most representative polyphenol component extracted from the rhizomes of *Curcuma longa* (known as turmeric). It represents 77 % of all curcumoids and “Generally Recognized as Safe” by the Food and Drugs Administration (FDA). According to the 2020 World Health Organization [48], low Covid-19 case fatality rate observed in South-East Asia and East-Mediterranean have been linked with dietary habits because curcumin is an integral part of spices used in food preparations in those regions. One of the recommendations of battling COVID-19 worldwide is reinforcing the immune system through nutritional supplementation and curcumin can be a promising candidate in protecting immunity. Numerous studies have been conducted to investigate the therapeutic effects of curcumin including its antiviral activity which was observed against several different viruses such as emerging arboviruses like the Zika virus (ZIKV) or chikungunya virus (CHIKV), hepatitis viruses, and respiratory influenza virus, herpes simplex virus-2, papilloma-virus as well as human immunodeficiency virus, human (HIV) [49–52]. Curcumin has a various way to exert antiviral effects, either through pathways crucial for normal virus function or through cellular processes [53,54] or directly on virus-encode factors [55,56]. For instance, it has been shown in a recent study that carbon quantum dots, from curcumin could boost antiviral effects of curcumin through different mechanisms against enterovirus 71 (EV71) in vitro and in vivo [57]. Furthermore, carbon quantum dots alone were found to be effective against the human coronas virus (HCoV) by inhibiting the entry receptor of HCoV-229E [58]. Curcumin poses electron transfer capable of scavenging various intracellular small oxidative molecules. In severe COVID-19 case, hypoxemia may happen due to pneumonia, which interferes with cell metabolism and increase anaerobic fermentation while reducing the energy supply [59]. Research has shown that curcumin, being a potent antioxidant, exerts its antioxidant effects by enhancing the production of antioxidant enzymes and neutralizing free radicals [60]. Curcumin (200mg/kg) increases the activities of superoxidase dismutase (SOD) level in acute injury induced by sepsis, and recovers the levels of xanthine oxidase(XO) and total antioxidative capacity (TAOC) while reducing the malondialdehyde (MDA) level in ventilator-induced lung injury in rats [61]. These studies reveal the antioxidant, anti-SARS-CoV-2, and potential immune-boosting effects of curcumin. Thus, curcumin could have a potential role in the prevention and control of COVID-19.

### Pharmacological Effects of Nigella sativa (Black Seed) against COVID-19

*Nigella sativa* (*N. sativa*) is one of the medicinal plants that have been used for centuries in most non-western cultures as part of traditional alternative treatments and remedies for various illnesses [62]. Extracts of *N. sativa* have therapeutic properties related to the alleviation of common symptoms of the novel COVID-19. For example, the aqueous extracts of *N. sativa* exhibit anodyne properties [63]. Similarly, the oil of *N. sativa*, exhibits anti-inflammatory properties in an experiment with rats [64]. Two biologically active agents from *N. sativa* (nigellimine and thymoquinone) have the potential considered to treat COVID-19 patients [65]. Nigellimine has similar biochemical structural properties and functional similarities with chloroquine and hydroxychloroquine which have been used in the treatment of COVID-19 [65]. The active component thymoquinone has been widely studied and attributed to most of the therapeutic effects of *N. sativa*. The activity of thymoquinone has been shown as an evident antiviral agent against avian influenza virus (H9N2) and murine cytomegalovirus infection model [66,67]. *Nigella sativa* possesses antiviral, antioxidant, anti-inflammatory, anticoagulant, immunomodulatory, antihistaminic, antitussive, antipyretic, and analgesic activities suggesting that it could be a potential medicinal drug to treat COVID-19 patients [68]. In addition, the active ingredients of *Nigella sativa* including nigellidine and –hederin have been identified as potential inhibitors of SARS CoV-2 [69].

### Pharmacological Effects of Vitamin D against COVID-19

**Figure 1:**
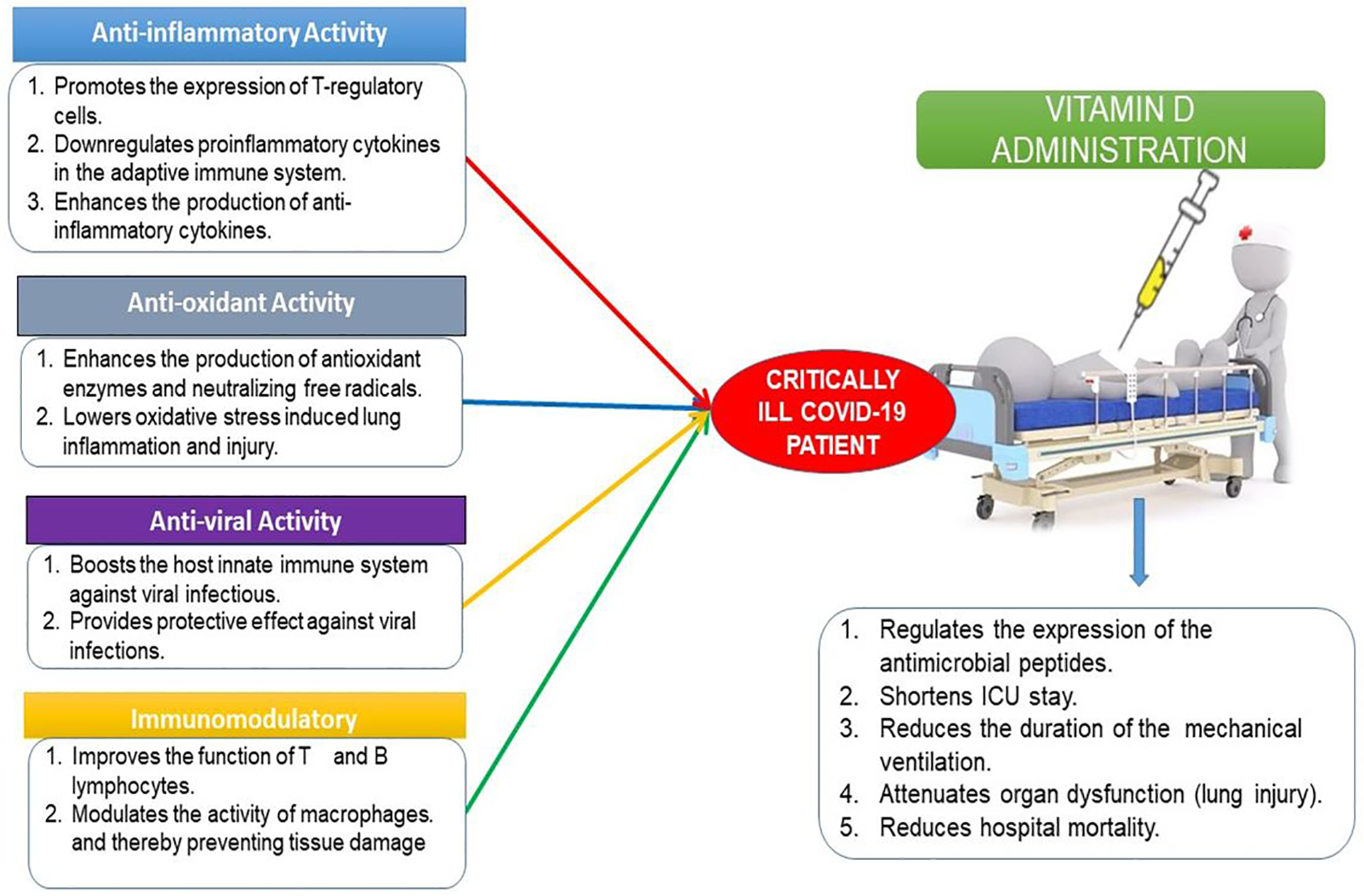
Schematic representation of vitamin D in the management of COVID-19. Vitamin D acts through several mechanisms of actions to prevent and control COVID-19 infection by forming physical barriers, boosting cellular natural immunity and adaptive immunity, and stimulating the release of antimicrobial peptides

Since the start of the COVID-19 pandemic, low levels of the vitamin D have been noted in association with severe COVID-19 infection. Vitamin D (calciferol) is a fat-soluble vitamin that plays a role in the enhancement of the immune system, regulation of bone growth, and absorption of calcium, iron, magnesium, phosphate, and zinc [70,71]. Vitamin D interferes with many immune system cells such as T lymphocytes, B lymphocytes, macrophages, neutrophils, and dendritic cells [72]. The T lymphocytes and B lymphocytes can act to form the active metabolite of vitamin D, 1,25(OH)2D3 which inhibits T cell activation and proliferation. In addition, vitamin D inhibits the production of pro-inflammatory cytokines and enhances the production of anti-inflammatory cytokines [72,73]. A scientific report showed that vitamin D inhibits the adaptive immune system and promotes the innate immune response which balances the immune response and provides an overall anti-inflammatory response [74], and in turn, immune cells differentially promote vitamin D metabolizing enzymes during infection^75^. Administration of vitamin D metabolites attenuates a variety of acute organ dysfunction, in particular, acute lung injury in animal models [76]. Zhang et al., [77] showed that vitamin D deficiency is associated with increased hospital mortality in critically ill adult patients. However, a randomized clinical trial suggested that administration of high dose vitamin D_3_ compared with placebo did not improve hospital length of stay and hospital mortality for vitamin D deficient (≤ 20 ng/mL) patients who are critically ill [78]. Vitamin D reduces viral respiratory infections, especially in people with low levels of vitamin D [72]. Since the beneficial effects of vitamin D in COVID-19 have been proposed, it has attracted more scientific investigations and it is currently in clinical trials for the prevention and treatment of COVID-19 [79,80]. A recent study identified vitamin as a potential chemopreventive agent against COVID-19 and influenza infections [81]. A clinical trial demonstrated that lower circulating levels of vitamin D metabolites are independently associated with worse outcomes in patients with acute illness, including COVID-19 patients [82]. COVID-19 is more prevalent among African Americans [83], older adults [84], nursing home residents and health care workers [85,86], and people with vitamin D deficiency [87]. However, it is less prevalent in pregnant women and young children [88]. Several convincing scientific studies have shown the association between vitamin D and more severe COVID-19 clinical manifestations [89–92]. Jain et al., [90] found that prevalence of vitamin D deficiency among patients with severe COVID-19 manifestations treated in intensive care units (ICU) was as high as 96.82%, in contrast to 32.96% in completely asymptomatic patients. One ecological study that included observation of the association between vitamin D deficiency and COVID-19 incidence, complications, and mortality in 46 countries found positive and statistically significant correction between vitamin D deficiency and all COVID-19 variables analyzed [91]. A study conducted by D’Avolio and colleagues demonstrated that 25-hydroxyvitamin D doses were lower in patients with positive for SARS-CoV-2 [93], suggesting the use of vitamin D supplementation as a useful strategy to lower the risk of infection. The therapeutic mechanisms of action by which vitamin D and its metabolites exert their effect include a range of biological responses involving immune system functions, processes in cellular growth and proliferation, and induction of apoptosis [94]. Vitamin D has been shown to regulate the expression of the antimicrobial peptides including human cathelicidin (LL-37), 1,25-dihdroxyvitamin, and defensins in a wide range of cell types including keratinocytes, epithelial cells, human monocytes and macrophages [95]. Cathelicidin (LL-37) is an antimicrobial peptide that eliminates intracellular mycobacteria and plays a role in the regulation of various processes of the autolysosomes [96]. LL-37 also has a pleiotropic role in a variety of biological responses, as a key immunomodulator with both pro-and anti-inflammatory functions in different cell lines and microenvironments in a context-dependent manner [95]. A series of scientific studies evaluated the effect of LL-37 on murine ligation and puncture (CLP) sepsis model and found that administration of LL-37 ameliorates the survival of CLP septic mice by suppressing the macrophage pyroptosis that induces the release of pro-inflammatory cytokines and augments inflammatory reactions in sepsis [97]. They also found that LL-37 induces the release of NETs with potent bactericidal activity and protects mice from CLP-induced sepsis [98]. Finally, they found that LL stimulates neutrophils to release antimicrobial ectosomes and improves murine sepsis [96]. Previous studies demonstrated that vitamin D downregulates pro-inflammatory cytokines such as interferon-γ, tumor necrosis factor-α, IL-1, IL-2, IL-6, IL-8, IL-12, T helper 1 cells, and B cells in the adaptive immune system [99]. At the same time, vitamin D appears to upregulate anti-inflammatory cytokines such as IL-4, IL-5, and IL-10. It also promotes the expression of T-regulatory cells, which turn off the adaptive immune response [73]. Figure 1 below summarizes the pharmacology and therapeutic use of vitamin D in the management of COVID-19.

### Pharmacological Effects of Zingiber officitale (Ginger) against COVID-19

Ginger is a natural root plant and a commonly consumed food ingredient asserted to have a range of different pharmacological properties [100,101]. Ginger contains several biologically active components mainly; gingerols and shogaols [100]. One of the active components of ginger (gingerol) has some potent anti-inflammatory action [102]. While COVID-19 is not primarily an inflammatory disease, the severity of the disease in some patients is attributable to the hyperinflammatory response of macrophages [103]. Hence, the anti-inflammatory role of ginger could prove useful in alleviating symptoms and disease severity in these cases. In addition, ginger has been shown to have therapeutic effects against metabolic diseases like diabetes and cardiovascular diseases in animal models [100]. This is relevant in relation to the COVID-19 because the mortality rates of COVID-19 positive patients are increased among people with comorbidities like diabetes, and cardiovascular diseases [100,104]. The observed antiviral potential of ginger is mediated through its antioxidant, immunomodulatory and anti-inflammatory ability [105]. The active compound allicin present in ginger is reported to have anti-influenza cytokines [106]. Ginger also has been shown to be an effective traditional remedy against common cold viruses [106]. 6-gingerol one of the active components of ginger has been shown to be an effective antiviral against the COVID-19 virus due to its high binding affinity with multiple binding sites of the viral protein molecules [107].

**Figure 2:**
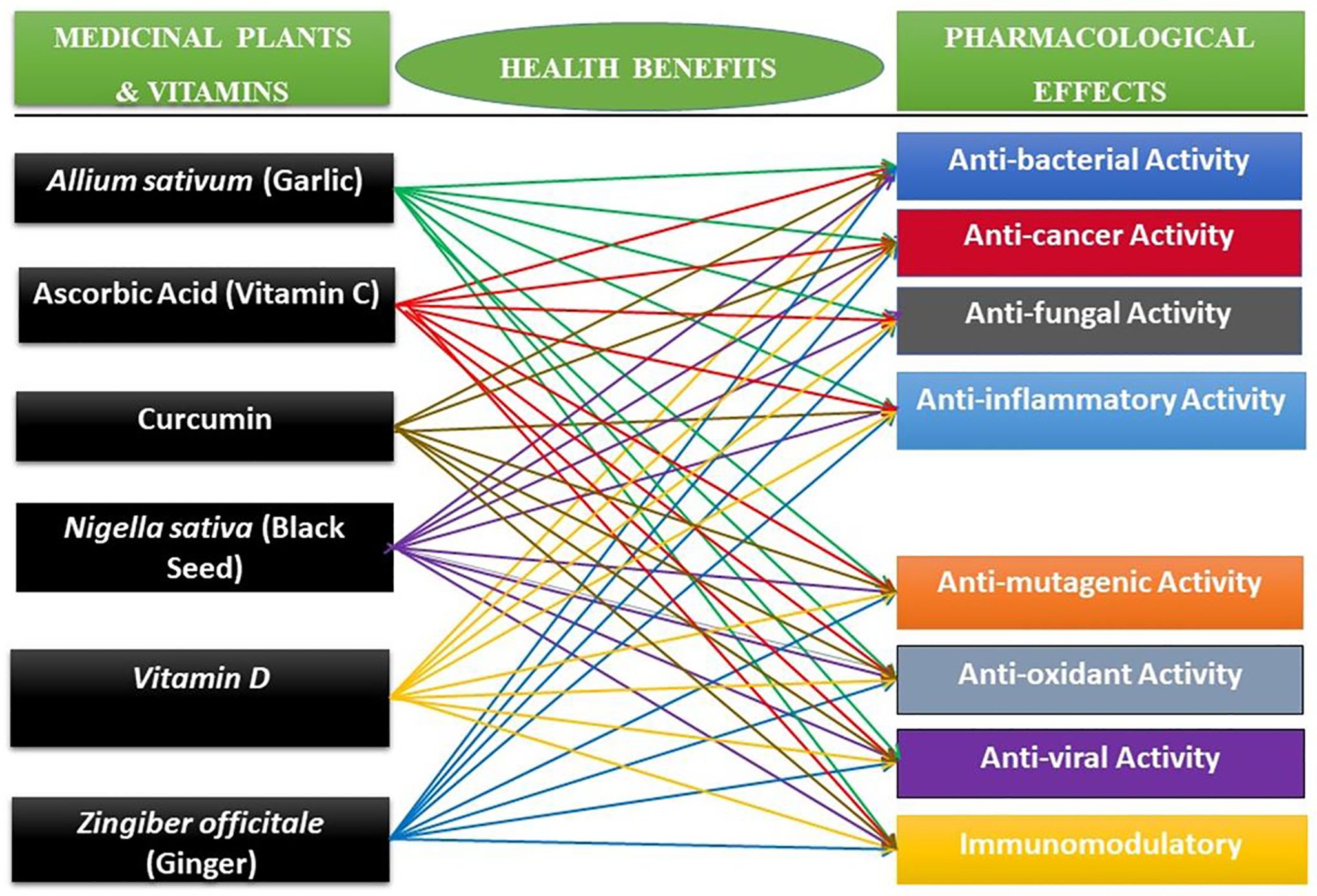
Summary of pharmacological effects of *Allium sativum*, ascorbic acid, curcumin, *Nigella sativa*, *Vitamin D, and Zingiber officitale*. The medicinal plants listed in Figure 2 are common herb consumed worldwide as a functional food and traditional home remedy for the prevention and/or treatment of infectious diseases which could benefit COVID-19 patients

Taken together, the literature search revealed that *Allium sativum*, ascorbic acid, curcumin, *Nigella sativa*, *Vitamin D, and Zingiber officitale* have in common eight (8) pharmacological effects including anti-bacterial, anti-cancer, anti-fungal, anti-inflammatory, anti-mutagenic, antioxidant, anti-viral, and immunomodulatory (Figure 2).

functional food and traditional home remedy for the prevention and/or treatment of infectious diseases which could benefit COVID-19 patients

Overall, medicinal plants with their long history of use in folk medicine for the treatment of infectious diseases have become a promising source of antimicrobial agents. They exhibit direct antimicrobial activities and/or indirect antimicrobial activities through synergism with antibiotics that increase their effectiveness. These medicinal plants and vitamins have potential effects in contoling SARS-CoV-2. However, preclinical, and clinical trials are needed to test for their uses for the treatment of COVID-19 patients. They exert their pharmacological effects on coronavirus disease through different mechanisms of action (Table 1).

## Conclusions

Various *in vitro* and *in vivo* studies, case reports, pilot studies, preclinical and clinical studies confirmed that *Allium sativum*, ascorbic acid, curcumin, *Nigella sativa*, Vitamin D*, and Zingiber officitale* possess in common anti-bacterial, anti-cancer, anti-fungal, anti-inflammatory, anti-mutagenic, antioxidant, anti-viral, and immunomodulatory activities related to the causative organism and symptoms of COVID-19. In the past, the world has experienced viral diseases in the form of epidemic or pandemic such as the severe acute respiratory syndrome coronavirus (SARS-CoV) outbreak in 2003, Middle East respiratory syndrome coronavirus (MERS-CoV) outbreak in 2012, an outbreak of Ebola disease in West Africa in 2014^112^, and currently COVID-19, an identified pathogenic illness that was first reported in Wuhan city, Hubei Province, China, on December 12, 2019. The clinical manifestations of COVID-19 vary from asymptomatic or paucisymptomatic forms to critical illness characterized by respiratory failure that warrants mechanical ventilation in an ICU^113^. Vitamin D and vitamin C have shown to be promising candidates for the treatment of COVID-19 in clinical practice. *Allium sativum*, curcumin, *Nigella sativa*, and *Zingiber officitale* are commonly consumed worldwide as functional food and traditional medicine for the prevention and/or treatment of infectious diseases including COVID-19 infection.

## Figures and Tables

**Table 1.** Mechanisms of action and clinical studies of *Allium sativum*, ascorbic acid, curcumin, *Nigella sativa*, vitamin D, and *Zingiber officitale* in the management of COVID-19

Medicinal Plants & Vitamins	Mechanisms of Action	Clinical Studies
*Allium Sativum* (garlic) 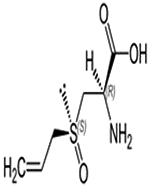	Garlic and its derivatives have potential antiviral activity against different human, animal, and plant pathogenic viruses through blocking viral entry into host cells, inhibiting viral RNA polymerase, reverse transcriptase, DNA synthesis and immediate-early gene 1(IEG1) transcription, and also through downregulation of the extracellular-signal-regulated kinase (ERK)/mitogen activated protein kinase (MAPK) signaling path^47^.	Clinical studies demonstrated the prophylactic and chemo preventive effects of garlic against viral infections in humans’ through enhancing the immune response^47^. A clin A clinical trial showed that supplementation of aged garlic modulated the inflammation and immunity of adults with obesity^108^.
Ascorbic acid 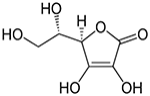	Ascorbic acid enhances the immune system by stimulating IFN production, lymphocyte proliferation, and enhancing the neutrophil phagocytic capability^109^.	A clinical trial demonstrated that vitamin C reduces pneumonia in lower respiratory tract infections. This clinical study suggest that vitamin C may work against SARS-CoV-2.
Curcumin 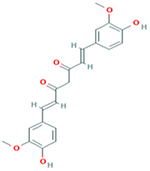	Curcumin has anti-inflammatory and anti-fibrotic effects by downregulating the expression of crucial chemokines and cytokines involved in lung infection such as INNy, MCP-1, IL-6^110^. Curcumin inhibits human respiratory syncytial virus infection by preventing RSV replication, release of TNF-alpha and downregulating phosphor-NF-kB^111^.	Clinical trials needed.
*Nigella Sativa* 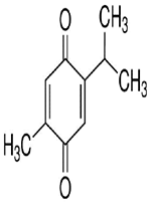	Two biologically active agents from *N. sativa* (nigellimine and thymoquinone) have the potential considered to treat COVID-19 patients^65^.	Clinical trials needed.
Vitamin D 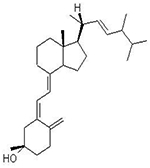	Immunomodulatory property^72^. Innate cellular immunity is strengthened by vitamin D actions directly or indirectly via antimicrobial peptides including human cathelicidin (LL-37), 1,25-dihdroxyvitamin, and defensins in a wide range of cell types including keratinocytes, epithelial cells, human monocytes and macrophages^95^.	Clinical studies looking at the impact of vitamin D on COVID-19 found that people who have vitamin D deficiency were more likely to test positive for the virus that causes COVID-19 compared to people with normal levels of vitamin D^90^.
*Zingiber Officitale* (Ginger) 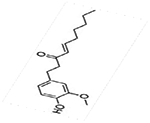	The active components of ginger (6-gingerol) have been shown to be an effective antiviral against the COVID-19 virus due to its high binding affinity with multiple binding sites of the viral protein molecules^107^.	Clinical trials needed.
